# Glucose-to-Fructose Isomerization: Optimisation and Mechanistic Insights Using Cheap Polyaluminium Chloride Catalyst

**DOI:** 10.3390/molecules31111803

**Published:** 2026-05-24

**Authors:** Antonella Angelini, Carlo Pastore

**Affiliations:** Italian National Research Council, Water Research Institute (IRSA-CNR), Viale Francesco De Blasio, 5, 70121 Bari, Italy; carlo.pastore@cnr.it

**Keywords:** Isomerization of glucose, polyaluminium chloride, benchtop NMR

## Abstract

The catalytic isomerization of glucose to fructose is a pivotal step in the valorisation of lignocellulosic biomass. In this study, polyaluminium chloride (PAC), a low-cost, industrially available material characterised by polynuclear aluminium–oxo species, is investigated for the first time as an alternative catalyst for this transformation. The catalytic performance of PAC was systematically investigated by varying the temperature (70–130 °C), solvent (pure water, H_2_O:MeOH 1:1 or 4:1) and reaction time (30–120 min). A fructose yield of up to 55% with a selectivity of 85% was obtained by using PAC at 120 °C in H_2_O:MeOH 1:1 for 120 min, confirming its effectiveness in promoting glucose isomerization to fructose. Mechanistic insights and quantitative monitoring were achieved using benchtop NMR spectroscopy. ^27^Al-NMR of PAC in aqueous solution exhibits two main signals at 1.12 ppm (attributed to the hexacoordinated Al complex) and at 63.3 ppm (associated with a tetrahedral Al centre typical of the Al_13_ Keggin-type cluster). With the increase in temperature, as well as by changing the reaction media from pure aqueous to a mixed aquo-alcoholic system, new Al species were generated that are more reactive than the starting AlCl_3_∙6H_2_O and PAC. Overall, this work demonstrates that PAC represents a viable, scalable, and more sustainable alternative to conventional aluminium-based catalysts, offering a promising route toward more efficient biomass conversion processes.

## 1. Introduction

The rapid and intense industrial growth of our society has progressively increased the exploitation of fossil resources for energy and fuel production. Given the limited natural availability of these resources, it has been necessary to focus on alternative, sustainable and widely available raw materials in order to meet the principles of a circular economy and mitigate the overexploitation of fossil-based feedstocks. In this context, industrial waste streams and lignocellulosic biomass represent promising low-cost resources that can be effectively valorised into high-value chemicals and biofuels [1,2,3].

**Figure 1:**
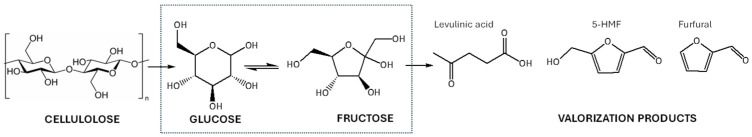
Steps for the conversion of cellulose into valuable products.

Most valorisation processes focus on the use of simple and complex carbohydrates, which have attracted significant interest over the past few decades due to their abundance and versatility. These compounds are often present in the side streams of industrial processes, such as those from the food, pulp and paper industries [4] and in waste sludge from urban wastewater treatment processes [5,6] and can also be readily obtained from lignocellulosic biomass through appropriate pretreatment and hydrolysis steps [7]. In particular, glucose derived from these sources can be isomerized into fructose, a key platform molecule for the synthesis of valuable derivatives such as 5-hydroxymethylfurfural (5-HMF), levulinic acid, and furfural. Therefore, glucose–fructose isomerization represents a crucial step in the efficient valorisation of renewable carbon resources (Figure 1) [8,9].

Several catalytic systems such as enzymes [10], bases [9,11] and Lewis acids [12,13,14,15,16] have been investigated to optimise the isomerization of glucose to fructose and enhance the valorisation of lignocellulosic feedstocks.

Although enzymatic catalysts exhibit excellent selectivity under mild conditions [10], their industrial implementation is hindered by high production and immobilisation costs, limited operational stability, and sensitivity to reaction conditions. Homogeneous bases and Lewis acids represent effective alternatives; however, several systems still suffer from limited selectivity, catalyst deactivation, corrosion issues, or difficult catalyst recovery [17].

The most recent literature reports the use of several highly active and stable heterogeneous catalysts, including doped zeolites, hydrotalcites, mixed metal oxides, biochar-supported catalysts, and metal–organic frameworks. Despite their promising catalytic performances, many of these materials require complex synthesis procedures, expensive supports, or high catalyst loadings, which may limit their large-scale implementation. In addition, catalyst deactivation, metal leaching, and surface fouling remain important challenges, particularly in biomass-derived real matrices [17].

Typically, heterogeneous catalysts are prepared by supporting species that are active in their homogeneous form (often metal chlorides) onto heterogeneous supports [18,19,20,21,22] while preserving the original catalytic mechanism [23,24].

This highlights the importance of developing new homogeneous systems that are not only catalytically efficient but also inexpensive, industrially available, and easily scalable. However, limited attention has been devoted to the selection of catalytic species in terms of production costs, industrial availability, and the possibility of recovery and reuse from existing industrial processes, despite the significant advantages associated with these aspects.

Among the homogeneous catalysts, AlCl_3_∙6H_2_O is one of the most common catalysts used and studied in glucose–fructose isomerization thanks to its low toxicity and good catalytic performance [13,25,26,27,28,29]. The catalytic mechanism and the nature of the real active species have been investigated in detail by Tang et al. [28] using electrospray ionisation tandem mass spectrometry (ESIMS/MS). Their study identified the cationic complex [Al(OH)_2_(H_2_O)]^+^ formed in situ under reaction conditions, as the real active species. This complex promotes glucose ring opening, coordinates the resulting acyclic form and facilitates the intramolecular 1,2-hydride shift leading to fructose formation. When alcohol is employed as the reaction medium, it competes with water for coordination with the aluminium centre, leading to the formation of alkoxy complexes [30]. These species were found to be even more catalytically active than their hydroxide counterparts. Such findings highlight the critical role of aluminium speciation in determining catalytic performance [31].

Despite its attractive catalytic properties, the industrial application of AlCl_3_∙6H_2_O is limited by its relatively expensive production process, which requires multiple crystallisation and water evaporation steps associated with high energy consumption and complex equipment [32].

Polyaluminium chloride (PAC) represents a particularly attractive candidate to overcome such drawbacks. PAC is a low-cost aluminium-based material widely produced at industrial scale for water-treatment applications and can also be recovered from industrial wastes and sewage sludge. Compared to conventional AlCl_3_·6H_2_O, PAC requires simpler production procedures with lower energy demand and reduced purification steps, making it potentially more suitable for large-scale applications within circular economy frameworks [33,34].

Moreover, PAC exists in aqueous solution as a dynamic mixture of monomeric, oligomeric, and polymeric aluminium species, including the well-known Al13 Keggin structure [35,36] whose transformation under reaction conditions may strongly influence catalytic activity. PAC can be prepared from low-cost raw materials such as bauxite [37] or recycled waste such as aluminium cans [38]. The process is often very easy and usually consists of treating the starting material with hydrochloric acid followed by titration with basification agents such as calcium oxide, sodium hydroxide or sodium aluminate, without needing water evaporation. Recently, strategies to recover PAC from sewage sludge have also been implemented. Various techniques such as acidification, basification, ion-exchanging, and electrodialysis have been used [39]. Considering that PAC is the most commonly used flocculant for water treatment in industrial production, with its usage reaching up to 3000–4000 tons per day [40,41], its recovery and reuse could represent an economically and environmentally attractive strategy.

In this work, the catalytic performance of PAC in glucose-to-fructose isomerization was systematically investigated and compared with that of conventional AlCl_3_∙6H_2_O. Particular attention was devoted to the influence of solvent composition, temperature, and pH on catalytic activity and aluminium speciation. In situ ^27^Al NMR spectroscopy was employed to gain mechanistic insights into the evolution of aluminium species under reaction conditions, while a benchtop NMR analytical protocol was developed for rapid quantitative monitoring of the reaction products.

To the best of our knowledge, this is the first study investigating PAC as a catalyst for glucose-to-fructose isomerization. Beyond evaluating catalytic activity, this work demonstrates the potential of low-cost industrial aluminium species as scalable catalysts for biomass valorisation and validates their applicability using a real industrial matrix derived from lactose-free milk production.

## 2. Results

Catalytic tests using PAC and AlCl_3_∙6H_2_O were carried out using glucose and a real matrix obtained as a by-product of lactose-free milk production. Sugar quantification was achieved using benchtop NMR spectroscopy after accurate validation of the quantitative method.

### 2.1. Quantitative Determination of the Reaction Products: Validation of the ^1^H-NMR Protocol

**Figure 2:**
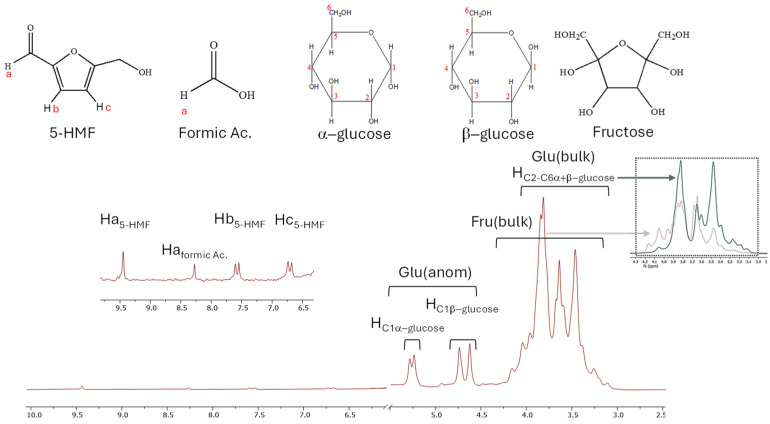
WET SUP-^1^H-NMR spectra of a mixture of glucose, fructose, 5-HMF and formic acid in acidic water.

In this work, a new protocol to quantify all reaction products in a single 20 min analysis performed directly in the reaction medium has been developed. The products of interest are glucose, fructose, 5-HMF and formic acid. 5-HMF and formic acid show isolated signals at 9.5 and 8.3 ppm, respectively, and can be easily quantified relative to an internal standard. The spectrum of glucose acquired on a 60 MHz benchtop NMR shows three main signals: a very broad, unresolved and complex signal in the region between 3.12 and 4.05 ppm (hereafter referred to as the bulk glucose glu(bulk), Figure 2) relative to protons from C2 to C6 and two doublets at 4.69 and 5.26 ppm (hereafter referred to as the anomeric glucose Glu(anom), Figure 2) relative to the C1 anomeric proton in α and β forms. The fructose spectrum shows a large signal between 4.17 and 3.26 ppm (hereafter referred to as Fru(bulk), Figure 2). In a mixture of glucose and fructose, the signals of the two sugars Glu(bulk) and Fru(bulk) overlap, making quantitative estimation of fructose difficult (Figure 2).

Moreover, the choice of solvent significantly affects NMR analysis, as its signals may overlap with those of the sugars, complicating spectral interpretation and integration. Solvent suppression can mitigate this effect, but if the solvent peak is too close to the analyte signals, distortions occur, compromising quantification by peak integration. For these reasons, different strategies need to be adopted depending on the solvent system.

**Figure 3:**
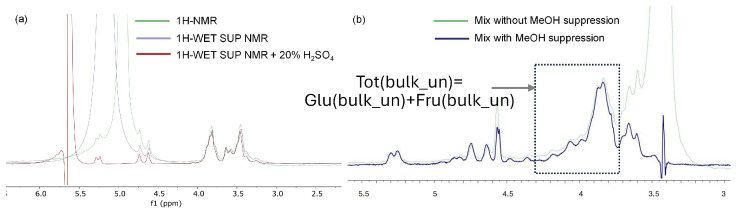
(**a**) ^1^H-NMR spectra of glucose in water using different protocols of acquisition; (**b**) ^1^H-NMR spectra of glucose–fructose mixture in methanol.

In water, the solvent signal at about 5 ppm masks the anomeric glucose resonances. Adding mineral acids, such as H_2_SO_4_, can shift the solvent signal and clear the area of interest (Figure 3a).

In methanol, the CH_3_ singlet at 3.4 ppm overlaps with the resonances of glucose and fructose. In this case, solvent suppression severely distorts the sugar peak profile in the region between 3.0 and 3.7 ppm, whereas the region from 3.7 to 4.3 ppm remains unaffected (Tot(Bulk_un), Figure 3b) and can be exploited for reliable sugar quantification.

On the basis of such findings, calibration curves were constructed using glucose–fructose mixtures by integrating the spectral region unaffected by solvent suppression. The contribution of glucose to this region was first estimated from the anomeric proton signals according to Equation (1):

Glu(bulk_un) = k · Glu(anom)(1)

where Glu(bulk_un) represents the area of the glucose bulk signal within the region unaffected by WET suppression, Glu(anom) is the total area of the glucose anomeric signals, and k is an experimentally determined constant. The value of k was obtained as the average of ten standard glucose solutions with concentrations ranging from 6 × 10^−3^ mg/mL to 60 mg/mL. The relative error associated with the determination of k was estimated to be 7%.

Subsequently, standard mixtures of glucose and fructose were analysed to construct the calibration curves. The fructose contribution was calculated by the difference between the total area of the unaffected bulk region and the glucose contribution (Equation (2)):

Fru(bulk_un) = Tot(bulk_un) − Glu(bulk_un)(2)

where Fru(bulk_un) is the area of the fructose bulk signal in the region unaffected by WET suppression, Tot(bulk_un) is the total bulk signal area in the same region and Glu(bulk_un) is the glucose contribution previously determined.

**Figure 4:**
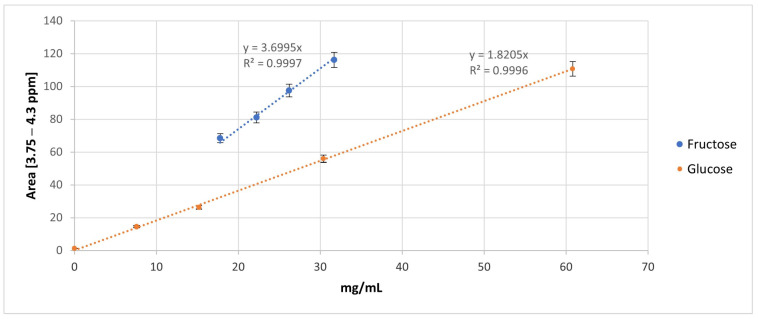
Glucose/fructose linearity curve constructed from ^1^H NMR signal area within the chemical shift in the range 3.75–4.3 ppm.

By plotting the integrated areas against the corresponding concentrations, the calibration curves shown in Figure 4 were obtained. Clear linear correlations were observed for both glucose and fructose (standard error of the slope SE = 0.1, R^2^ > 0.999).

Similarly, the formation of fructose from lactose (galactose + glucose) present in the real matrix was assessed by calculating the area of the fructose signals as the difference between the total integrated area in the region from 3.7 to 4.3 ppm and the area corresponding to lactose, estimated based on the relative intensities of the anomeric proton signals.

Trueness was assessed by comparing the ^1^H-NMR results with those obtained by HPLC quantification using 10 standard solutions of glucose and fructose.

**Figure 5:**
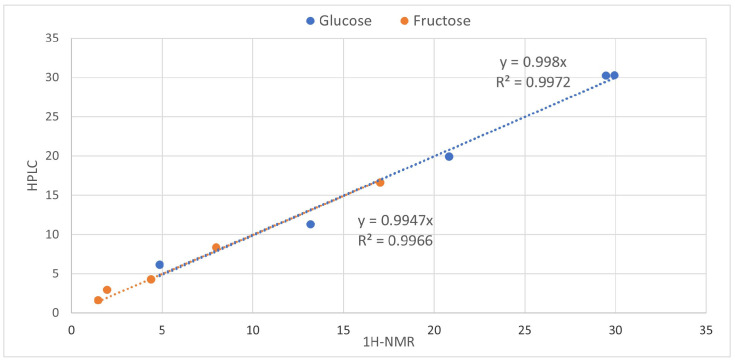
Correlation plot of glucose and fructose concentrations obtained by ^1^H-NMR versus obtained by HPLC.

Figure 5 presents the correlation plot of the NMR results versus concentrations obtained from the chromatographic method. Deviations of the data points from the diagonal lines indicate concentration differences. A linear regression constrained to pass through the origin was performed, yielding the slope and the correlation coefficient r. An almost perfect agreement was observed across the concentration range from 1 to approximately 30 mg/mL (correlation coefficient r > 0.99, standard error of the slope SE = 0.06). These results confirm the suitability of the NMR method for determining the concentrations of individual components.

### 2.2. Catalytic Glucose-to-Fructose Isomerization over AlCl_3_·6H_2_O, PAC and PAC/NaOH

#### 2.2.1. Reaction Monitoring in Aqueous Medium

The catalytic activity of PAC and AlCl_3_∙6H_2_O was compared in aqueous medium in a range of temperatures between 70 and 130 °C. The activity of each catalyst was estimated based on glucose conversion, fructose yield, and selectivity. The resulting data obtained after 30 min of reaction are summarised in Table 1.

**Figure 6:**
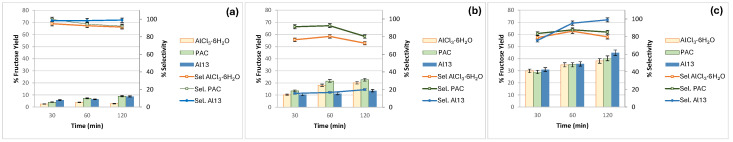
Glucose–fructose isomerization in aqueous medium. Reaction conditions: VH_2_O = 5 mL, m(glucose) = 225 mg, m(catalyst) = 30 mg. Time-dependent profiles at (**a**) 90 °C, (**b**) 120 °C, and (**c**) 130 °C.

No detectable conversion was observed for either catalyst below 90 °C. At 120 °C, all catalysts give fructose as the main product with mannose present as a secondary product (<0.5%). AlCl_3_·6H_2_O provided a glucose conversion of 13.4% and a fructose yield of 10.2%, corresponding to a selectivity of 76.5%, in good agreement with data in the literature [28]. PAC showed slightly higher fructose yield (13.5%) and better selectivity (91.32%) under identical conditions, suggesting a moderate improvement in catalytic activity compared with AlCl_3_·6H_2_O. Such a trend is also preserved at longer reaction times (Figure 6).

Raising the temperature to 130 °C, the fructose yield obtained with PAC reached 28.9% after 30 min and about 40% after 2 h; however, the selectivity began to decrease (from 91% to 83%) due to the enhanced rate of secondary reactions. It is known that higher temperatures promote the production of mannose (3.7%) and the dehydration of hexoses to 5-hydroxymethylfurfural (0.2%) and subsequent degradation products, such as formic and levulinic acids, as widely reported for AlCl_3_-based catalytic systems in aqueous media [28].

The data in the literature indicate that the speciation of aluminium in aqueous solution is strongly dependent on pH and the solvent used. In the case of PAC, specific structures can form under controlled pH conditions, including the well-known Keggin-type Al_13_ polycation, which consists of clusters containing 13 aluminium atoms [35,36]. To investigate the catalytic activity of PAC under these structural conditions, the pH of the reaction mixture was adjusted with NaOH until a stable pH of 4.3 was reached. At this pH, PAC exists as a mixture of oligomeric clusters and monomeric species, as confirmed by ^27^Al NMR analysis (see below).

The catalytic behaviour of this modified PAC sample (denoted as Al_13_ in Table 1 and Figure 5) differed significantly at 120 °C: the fructose yield was lower, and the selectivity decreased to approximately 16% after 30 min. However, increasing the temperature to 130 °C led to fructose yields and an overall catalytic performance comparable to those obtained with the other catalysts. At the same time, the pH returned to more acidic values (pH = 3.2, the value at room temperature before the addition of the NaOH mixture). These results suggest that aluminium speciation plays a critical role in determining catalytic efficiency, highlighting the importance of the structural features of the active species in glucose isomerization.

#### 2.2.2. Reaction Monitoring in Alcoholic Medium

Many studies reported that glucose isomerization to fructose can be achieved in an alcoholic medium via the formation of fructoside (pyranoside and furanoside) species, which shift the reaction equilibrium toward the products, thereby enhancing both the reaction rate and product yield [17,42,43].

**Figure 7:**
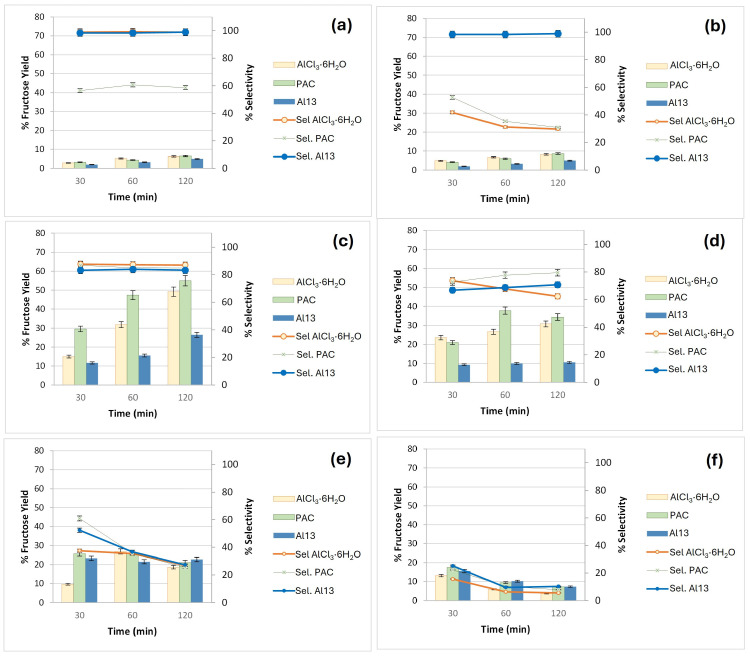
Glucose–fructose isomerization in water/methanol mixtures under different conditions. Reaction conditions: m(glucose) = 225 mg, m(catalyst) = 30 mg. (**a**,**c**,**e**) H_2_O/MeOH = 1:1 (*v*/*v*; 2.5 mL/2.5 mL) at 90, 120, and 130 °C, respectively; (**b**,**d**,**f**) H_2_O/MeOH = 1:4 (*v*/*v*; 1.0 mL/4.0 mL) at 90, 120, and 130 °C, respectively.

In this work, we tested the catalyst in the presence of methanol, the simplest and most representative alcohol, allowing the reaction to be conveniently monitored by NMR. The catalytic performances of AlCl_3_·6H_2_O, PAC and PAC combined with NaOH (Al_13_) were investigated in water/methanol mixtures over a temperature range of 90–130 °C (Figure 7).

**Figure 8:**
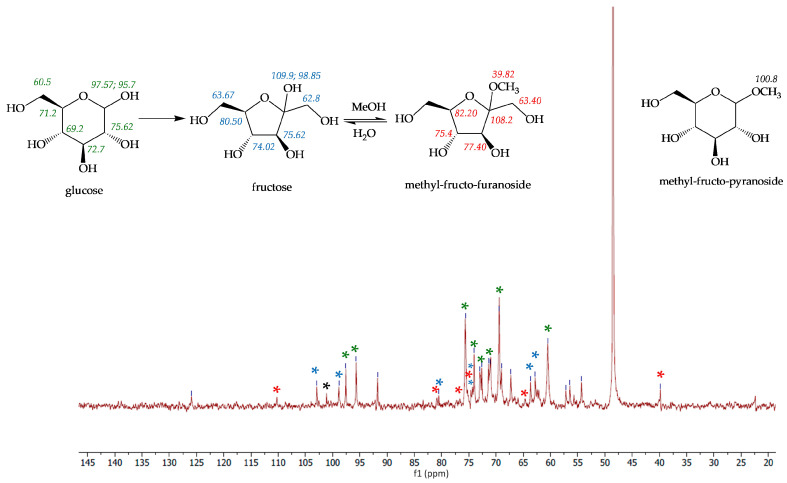
^13^C NMR spectrum of reaction mixture (reaction time = 30 min, T = 120 °C, H_2_O = 2.5 mL, MeOH = 2.5 mL, m(glucose) = 225 mg, m(catalyst) = 30 mg). Signals marked with the green asterisk (*) correspond to glucose, those in blue to fructose, those in red to methyl-fructo-furanoside and those in black to methyl-fructo-pyranoside.

The results show that the presence of methanol increases fructose yield compared to a purely aqueous medium. The highest yields were observed at 120 °C in a H_2_O:MeOH mixture of 1:1 (*v*/*v*). Under these conditions, PAC exhibited significantly higher catalytic activity than AlCl_3_·6H_2_O, in agreement with the trend already observed in water: PAC afforded a fructose yield of 29.63% after 30 min, compared to 15% obtained with AlCl_3_·6H_2_O. At longer reaction times, this difference in activity became less pronounced. An excess of methanol relative to water (H_2_O:MeOH = 4:1 *v*/*v*; Figure 7b,d,f) led to lower fructose yields and selectivity for both catalysts. This behaviour can be rationalised based on the reaction mechanism reported in the literature: in the presence of alcohols, glucose is converted into fructose that rapidly forms alkyl fructosides [31,44,45]. These intermediates are subsequently hydrolysed in an aqueous medium to yield fructose. Evidence supporting this mechanism was obtained from ^13^C NMR analysis, which clearly shows the presence of unconverted glucose, fructose, and methyl fructoside in both furanoside and pyranoside forms (Figure 8).

Most probably, the rate of hydrolysis depends on the amount of water present; therefore, in MeOH:H_2_O 4:1 *v*/*v* mixtures, the conversion of fructosides to fructose is slower, resulting in lower fructose yields with respect to that obtained in MeOH:H_2_O 1:1 *v*/*v* mixtures. For the same reason, selectivity is also reduced under these conditions, as alkylated sugar derivatives contribute to the overall product distribution.

Several studies in the literature have reported that improved results for glucose–fructose isomerization can be achieved through a two-step process: the first step is carried out in an alcohol medium to form alkyl fructosides, followed by a second step in water to promote their hydrolysis to fructose [45]. However, considering that PAC is typically synthesised and used in aqueous solution, and that many real glucose-containing feedstocks are also water-based, this approach was not investigated in the present work.

### 2.3. In Situ ^27^Al NMR Study: Influence of Solvent, pH and Temperature on Aluminium Speciation

In order to study the speciation of the catalyst during the glucose–fructose isomerization, in situ ^27^Al NMR experiments were performed throughout the reaction process.

**Figure 9:**
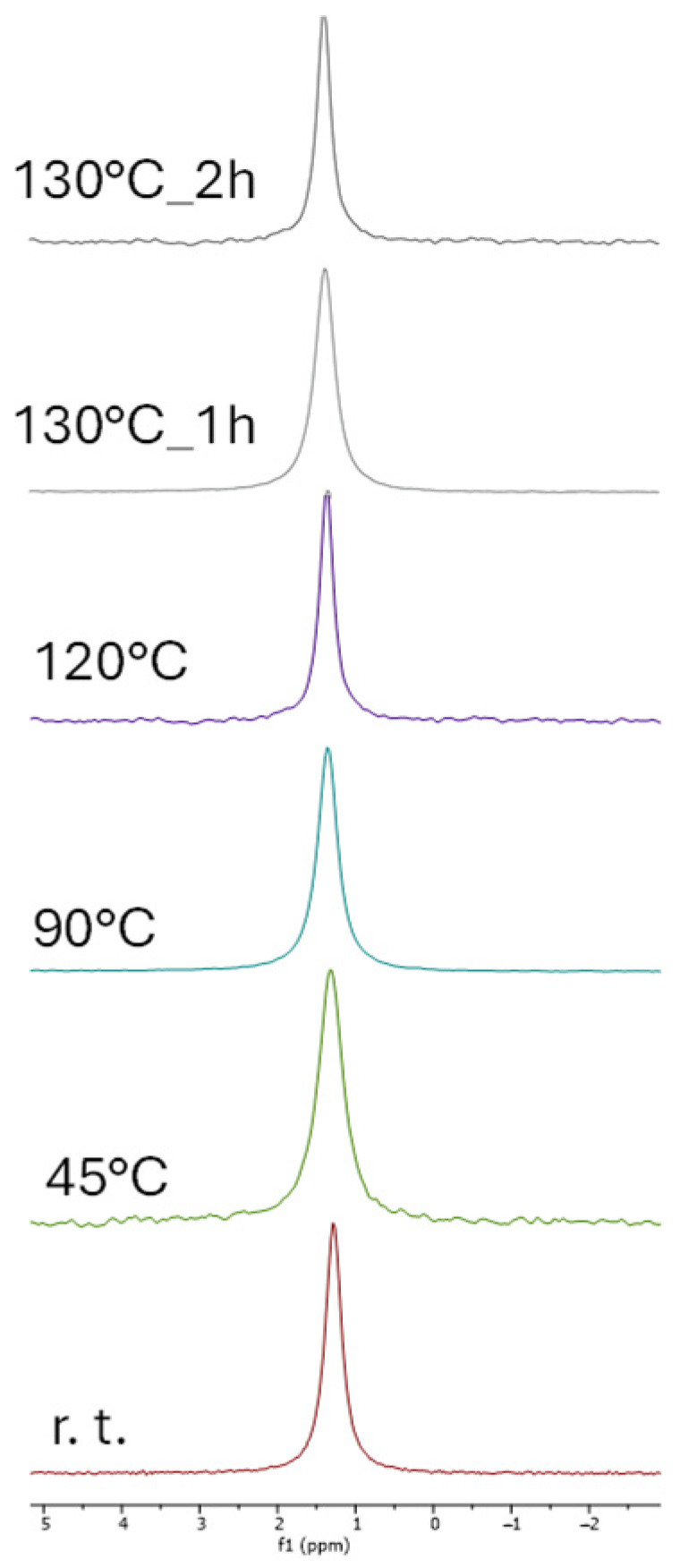
^27^Al NMR spectra of AlCl_3_·6H_2_O in aqueous solution over the temperature range 25–130 °C.

AlCl_3_·6H_2_O exhibits a sharp singlet at 1.12 ppm, which can be attributed to the well-known monomeric hexacoordinated aluminium species, which is stable from room temperature up to 130 °C (Figure 9) [28].

In alcoholic media, no significant shift in the resonance is observed in the ^27^Al NMR spectrum, which is essentially superimposable with that recorded in water. This behaviour can be explained by the coordination of alcohol molecules with the aluminium centre, leading to analogous hexacoordinated monomeric complexes identified at 1.12 ppm. The chemical environment of the aluminium species is preserved up to 130 °C.

^27^Al NMR of PAC in aqueous solution exhibits two main ^27^Al NMR signals: a resonance at 1.12 ppm attributed to the monomeric hexacoordinated complex, and a resonance at 63.3 ppm associated with the Al_13_ Keggin-type cluster. The data in the literature demonstrate that the latter consists of a central Al^3+^ ion coordinated to four oxygen atoms and surrounded by twelve octahedrally coordinated Al^3+^ cations connected through µ_2_-OH bridges [46]. Additional aluminium species within the Keggin structure possess distorted geometries that lead to very broad and often undetectable NMR signals. It is worth noting that the signal at 1.12 ppm compared with that of AlCl_3_ in water appears larger, covering the region from 5 to −5 ppm. This suggests the presence of different Al complexes, possibly containing multiple Al nuclei, which could be prone to coordinating the sugar.

**Figure 10:**
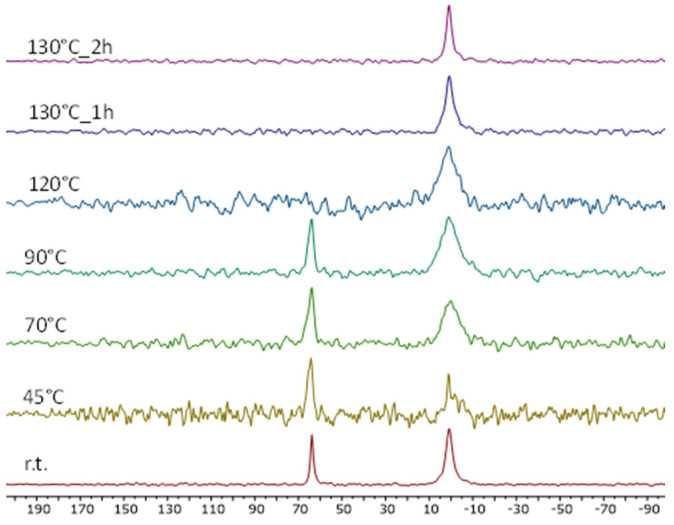
^27^Al NMR spectra of PAC in aqueous solution over the temperature range 25–130 °C.

Figure 10 shows the evolution of the ^27^Al NMR spectrum of PAC (polyaluminium chloride) in aqueous solution as a function of temperature. The pH was initially adjusted to 4.5 to promote the formation of the Al_13_ Keggin-type species. Upon increasing the temperature, significant spectral changes were observed. At 45 °C, the broad signals in the high-field region began to decrease, likely due to the coordination of solvent molecules and glucose to the Al centres, leading to the formation of species undetectable on the NMR scale. In contrast, the signal at 63.3 ppm, attributed to the Al_13_ Keggin structure, showed only a slight decrease, consistent with its relatively high stability and reduced accessibility of the metal centres to ligands such as water or glucose.

At 70 °C, the signal at 63.3 ppm developed a shoulder at a lower chemical shift, while the resonance centred around 1.12 ppm became significantly broader, indicating the presence of multiple aluminium environments. At 90 °C, corresponding to the onset of glucose conversion, the intensity of the 63.3 ppm signal further decreased, whereas the broad signal around 1.12 ppm increased, suggesting the progressive transformation of the Keggin species into more reactive forms.

**Figure 11:**
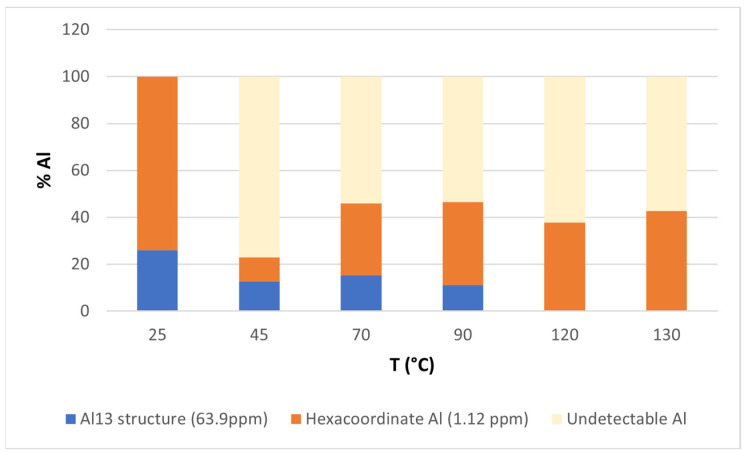
Evolution of aluminium speciation as a function of temperature (25–130 °C).

At 120 °C, where the high catalytic activity is observed, the signal at 63.3 ppm disappeared completely, while the broad resonance around 1.12 ppm dominated the spectrum, consistent with the presence of a mixture of mononuclear and polynuclear aluminium species. Notably, the sum of the intensities of the signals at 63.3 and 1.12 ppm was not conserved during heating, indicating that the Al_13_ Keggin structure does not simply convert into the observable hexacoordinated monomeric species. Instead, it likely evolves into a range of colloidal polynuclear oligomer highly distorted aluminium species that are not detectable by ^27^Al NMR (Figure 11) [46].

**Figure 12:**
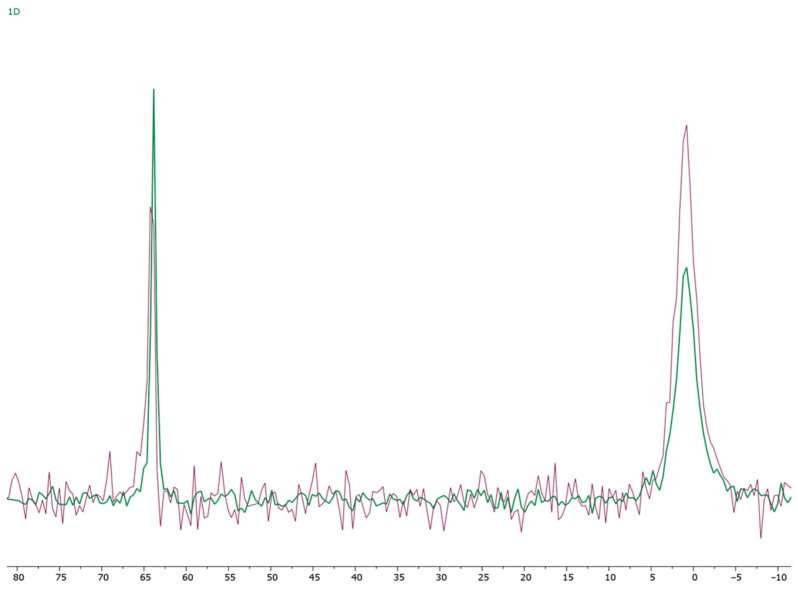
^27^Al NMR spectra of PAC in H_2_O (fuchsia) and H_2_O:MeOH 1:1 *v*/*v* (green) at room temperature.

In the presence of methanol, the alcohol molecule replaces the ligated aqua ligand and destabilises the tridecamer Al_13_ species, which happens even at room temperature (Figure 12), promoting the formation of the actual active species (Figure 12) [47].

The faster formation of the active species from PAC compared to AlCl_3_·6H_2_O is also supported by the recorded T_1_ values. (Table 2) Specifically, the T_1_ relaxation time of glucose in water (1.57 s) remains unchanged when glucose is added to an AlCl_3_∙6H_2_O–water system from room temperature up to 120 °C, at which point it drops sharply to 0.98 s, indicating a more dynamic molecular environment and stronger interactions. In contrast, when glucose is added to a PAC–water mixture, the T_1_ values decrease from 1.48 s to 0.9 s even before 90 °C is reached and remain nearly constant up to 120 °C (0.8 s).

The T1 values do not show a direct quantitative correlation with fructose yield but they provide qualitative information about the dynamic interactions occurring between glucose and the aluminium species under reaction conditions. In the case of PAC, the decrease in T1 already observed at 90 °C suggests the presence of a more dynamic coordination environment, likely associated with the coexistence of different aluminium species. This species can interact with glucose and destabilise its structure, resulting in increased molecular mobility and reduced relaxation times, even though fructose formation remains limited at this temperature. The catalytic conversion depends not only on substrate coordination, but also on the formation of sufficiently reactive catalytic species able to efficiently promote the hydride-shift isomerization step. Therefore, although the lower T1 values indicate enhanced glucose–aluminium interactions and a less stable glucose environment, the concentration of the real active species remains insufficient to achieve high fructose yields.

### 2.4. Glucose–Fructose Isomeration in a Real Matrix with AlCl_3_∙H_2_O and PAC

Wastewater from lactose-free milk production contains a high concentration (9.9 wt%) of hydrolysed lactose, consisting of a mixture of glucose and galactose. This stream was selected as a representative real matrix to evaluate the catalytic performance of PAC in glucose isomerization.

The reaction conditions were selected based on results from model glucose solutions. Upon addition of PAC to the real matrix, a white precipitate formed. This behaviour can be attributed to the well-known coagulation properties of PAC, which is commonly employed as a flocculant in wastewater treatment [48]. As a consequence, a higher amount of PAC (approximately 50% more) was required to ensure an equivalent concentration of catalytically active aluminium species in solution. In particular, after the addition of PAC, the white solid was removed by centrifugation, and the amount of Al in solution was determined by Al-qNMR (see Experimental Section). Then, the required amount of PAC was added in order to reach the equivalent amount of metal in solution with respect to the experiment performed with AlCl_3_·6H_2_O. The experiments were carried out at 120 °C for 2 h. The catalytic performance of PAC was compared with that of AlCl_3_·6H_2_O, and the corresponding results are summarised in Table 3.

The catalytic performance observed in the real matrix was consistent with that obtained using standard glucose solutions.

## 3. Discussion

### 3.1. Quantitative Determination of the Reaction Products Through ^1^H NMR

The ad hoc benchtop ^1^H NMR protocol developed in this work to monitor the isomerization of glucose to fructose represents a valid and competitive alternative to the most widely used analytical approaches for sugar quantification. It allows the simultaneous quantification of a wide range of products not limited to sugars, namely, 5-HMF and formic acid, within a single acquisition of approximately 20 min, just by adding the proper internal standard.

Compared to the most popular HPLC and IC techniques [7,49], this protocol offers several advantages: it is non-destructive, requires minimal sample preparation, avoids dilution steps that could introduce additional uncertainties and, in contrast to Gas Chromatography, does not require any derivatization [50,51].

Although benchtop NMR systems are characterised by lower sensitivity and spectral resolution compared to high-field instruments, recent advances in solvent suppression techniques, combined with chemical shift modulation by acid addition, enable spectra to be acquired directly from the reaction mixture without the use of deuterated solvents. This significantly simplifies the analytical procedure and enhances its applicability to real-time reaction monitoring.

The trueness of the method was verified by comparison with a conventional HPLC technique (correlation coefficient r > 0.99), supporting the effectiveness of benchtop NMR spectroscopy as a rapid, reliable, and practical quantitative tool for monitoring sugar isomerization processes.

### 3.2. Catalytic Glucose-to-Fructose Isomerization over PAC

The results obtained in this study demonstrate that polyaluminium chloride (PAC) is an effective catalyst for glucose-to-fructose isomerization and represents a viable alternative to the more expensive AlCl_3_·6H_2_O. Under optimised conditions, PAC provided comparable or even higher fructose yields and selectivity than the conventional aluminium chloride catalyst. In particular, the higher selectivity observed at 120 °C suggests that PAC promotes glucose isomerization while partially limiting secondary degradation reactions leading to by-products such as HMF and organic acids.

The comparison between PAC and AlCl_3_·6H_2_O clearly indicates that catalytic activity is not simply related to the total aluminium concentration, but rather to the nature and evolution of aluminium species under reaction conditions. Combining catalytic experiments with ^27^Al NMR analysis, it was observed that PAC undergoes a temperature- and pH-dependent transformation from well-defined Al_13_ Keggin-type clusters (63.3 ppm) to a broader distribution of mono- and oligomeric aluminium species centred around 1.12 ppm. At 90 °C, PAC consists of a complex equilibrium involving Al_13_ clusters, oligomeric species, and undetectable colloidal forms [46]. Among these, Al_13_ is catalytically inactive due to its closed structure, which limits substrate coordination, whereas monomeric and dimeric species represent the active forms, with colloidal species likely contributing to the activity thanks to the highly distorted structure that could favour the coordination of glucose.

Upon increasing the temperature to 120 °C, the Al_13_ species disappear, and the system is dominated by oligonuclear aluminium species, which are both catalytically active and exhibit the highest selectivity toward fructose formation. At 130 °C, the ^27^Al NMR spectrum shows a sharp signal characteristic of monomeric species, corresponding to catalytic behaviour comparable to that of AlCl_3_·6H_2_O.

These observations indicate that Al_13_ does not act as the active species, but rather as a precursor that undergoes thermal transformation into smaller, more reactive aluminium species above 45 °C. Indeed, if NaOH is added to the reaction mixture in order to increase the amount of Al in the kegging structure (Al_13_ in Table 1), PAC is less active and a very low selectivity is observed even at 120 °C: under these conditions fructose formation proceeds slowly through the contribution of the limited fraction of active aluminium species. At the same time, the alkaline conditions introduced by NaOH and the high temperature promote glucose degradation pathways, leading to undesired by-products, resulting in the particularly low selectivity observed at 120 °C [52,53]. At 130 °C, however, the Al_13_ Keggin-type species undergo thermal transformation into smaller and more catalytically active mono- and oligomeric aluminium species, as supported by the ^27^Al NMR results discussed in the manuscript. Under these conditions, glucose-to-fructose isomerization becomes kinetically competitive with glucose degradation reactions, leading to a substantial improvement in fructose selectivity.

The enhanced catalytic performance is therefore associated with the formation of accessible, dynamically coordinated mono- and oligomeric aluminium complexes capable of facilitating glucose coordination and hydride transfer more efficiently than the relatively stable monomeric complexes generated from AlCl_3_·6H_2_O. These findings are consistent with theoretical studies reported in the literature, which indicate that dimeric complexes can catalyse the reaction more efficiently than monomeric catalysts, as observed, among others, in typical enzymes that isomerise glucose to fructose with high selectivity [54]. However, such species are not formed from AlCl_3_·6H_2_O, most probably because the energy required for dimeric complex formation is high, since the metal centres repel each other and it is entropically less favourable for glucose to interact with two metal cations rather than with a single one [15].

The effect of the solvent further confirms the key role of coordination chemistry in the reaction mechanism. The presence of methanol significantly enhances fructose yield, particularly in H_2_O 1:1 mixtures, likely because alcohol molecules promote the formation of reactive alkoxy-aluminium species that facilitate glucose isomerization and stabilise fructoside intermediates. However, excessive methanol content decreases fructose selectivity, probably due to slower hydrolysis of alkyl fructosides and the accumulation of side products.

The experiments performed on the real matrix derived from lactose-free milk production demonstrate that PAC maintains its catalytic activity even in complex systems, confirming the robustness and practical applicability of the system. Nevertheless, the intrinsic coagulation properties of PAC caused partial precipitation phenomena, reducing the concentration of soluble active species and requiring higher catalyst loading. This aspect highlights a potential limitation that should be considered for future process optimisation and scale-up.

Compared with several catalytic systems reported in the literature, PAC offers important practical advantages, including low production cost, direct industrial availability, simpler preparation procedures, and the possibility of recovery from industrial waste streams and sewage sludge. These characteristics make PAC particularly attractive for large-scale biomass valorisation processes within circular economy frameworks.

By matching the catalytic performance with the aluminium speciation and the coordination environment, the present study provides useful guidelines for the design of more efficient and sustainable heterogeneous catalytic systems for biomass valorisation in the future.

## 4. Materials and Methods

All reagents, organic solvents, and standards were Sigma-Aldrich pure-grade reagents (99%).

**Figure 13:**
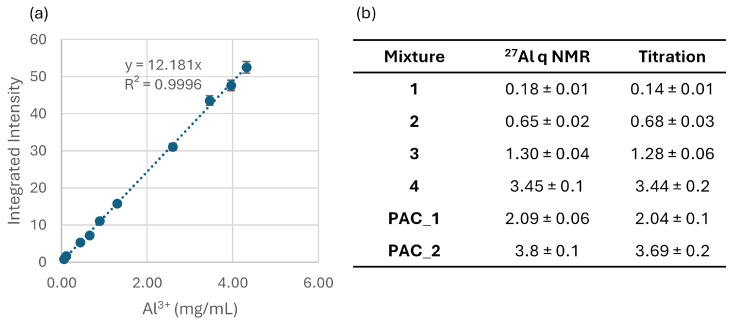
^27^Al NMR calibration curve and validation (**a**) through titration method (table (**b**)).

Polyaluminium chloride (PAC) was purchased from Mediterranean Chemical Company s.r.l. (Bari, Italy) The total aluminium content was determined by ^27^Al-qNMR and verified by complexometric back-titration. For NMR analysis, a calibration curve was generated using an external standard approach: Al^3+^ solutions were prepared by dissolving AlCl_3_·6H_2_O in water. The acquired ^27^Al NMR spectra displayed a single resonance attributed to the hexacoordinated species. Excellent linearity (error of the slope SE = 0.1, R^2^ = 0.996) was achieved over a broad concentration range (0.05–4.5 mg mL^−1^) (Figure 13).

The quantification was validated by determining the Al content of selected calibration solutions via complexometric titration. A known excess of EDTA was added to the sample, and the pH was adjusted to approximately 10 with ammoniacal buffer. The solution was heated briefly to ensure complete complexation, cooled, and then back-titrated with standardised ZnSO_4_ solution using Eriochrome Black T as the indicator, with the endpoint detected by the colour transition from blue to wine-red [55]. The two analytical methods produced consistent results (Table in Figure 13b).

For the determination of the aluminium content in PAC, a weighed amount of sample was dissolved in water and treated with an excess of NaOH to convert all Al ions into the hydroxide form, ensuring that no NMR-invisible aluminium species remained. The measured total Al content was 9.5% by NMR and 9.24% by titration.

All catalytic experiments were repeated at least in triplicate under identical conditions, and the reported values of glucose conversion, fructose yield, and selectivity are now presented as mean values with the corresponding standard deviations. Error bars have also been added to the relevant figures and tables where appropriate.

In a typical catalytic experiment, 0.225 g of glucose and 30 mg of AlCl_3_·6H_2_O (or 36 mg of PAC to provide an equivalent amount of Al) were dissolved in 5 mL of deionized water, or alcoholic mixtures. Reactions were conducted at the desired temperature (70–130 °C) for 2 h in a glass reactor.

The composition of the reaction mixtures was monitored by ^1^H, ^13^C and ^27^Al. NMR was performed on a benchtop Spinsolve Ultra 60 MHz NMR spectrometer (Magritek, Aachen, Germany).Shimming was performed according to the manufacturer guidelines with a mixture of 5% H_2_O and 95% D_2_O. The temperature of the magnet was controlled at 20 °C. The sample parameters for the ^1^H NMR analysis were as follows: 32 scans, an acquisition time of 3.2 s, a repetition time of 10 s and a pulse angle of 90°, with the ^13^C decoupler on. For the ^27^Al measurements the following acquisition parameters were set: 2048 scans, a repetition time of 100 ms, an acquisition time of 77 ms, a pulse angle of 45°, and a centre frequency of 0 ppm.

The NMR quantification was validated by comparison with high-performance liquid chromatography (HPLC) using a JASCO system (Mary’s Ct, Easton, MD, USA) equipped with a Hi-Plex H column (300 × 4 mm; Agilent, Santa Clara, CA, USA) and two detectors: an RI-150 refractive index detector and a UV-150 detector operated at 235 and 260 nm. Sample injection was performed automatically (100 µL) using an AS-2055 autosampler. The column was maintained at 55 °C, and the mobile phase consisted of 0.01 M H_2_SO_4_ at a flow rate of 0.6 mL min^−1^.

Tests on a real matrix were performed using a serum derived from lactose-free milk production, provided by Granarolo company (Gioia del Colle, Italy). The serum was fully characterised, showing a total sugar content of 11.4 wt% (galactose: 7.5 wt%; glucose: 3.9 wt%), total solids (TS) of 22.02 wt%, and inorganic salts including Na (0.3 wt%) and K (0.4 wt%), as determined by ICP-MS.

## 5. Conclusions

In this work, polyaluminium chloride (PAC) has been demonstrated to be a promising alternative catalyst for the isomerization of glucose to fructose, providing catalytic performances comparable to those of AlCl_3_·6H_2_O and, under optimised conditions, higher selectivity.

^27^Al NMR analysis revealed that the catalytically active species are not the well-defined Al_13_ Keggin-type clusters, but rather smaller, more accessible mono- and oligomeric aluminium species generated upon heating. The transformation of Al_13_ into these active forms above 45 °C is essential to achieve high catalytic efficiency, as these species provide the accessible coordination sites necessary for substrate activation and hydride shift.

Importantly, the applicability of PAC was successfully demonstrated in a real industrial matrix derived from lactose-free milk production, confirming the robustness of the catalytic system under complex conditions. This aspect, combined with PAC’s low cost and low toxicity, supports its potential for practical implementation in circular economy frameworks.

Finally, this work introduces an innovative analytical approach based on benchtop NMR, which proved to be a powerful and accessible tool for monitoring reaction progress and probing catalyst speciation in real time. Overall, these findings provide new insights into the design of aluminium-based catalytic systems and contribute to the development of efficient, sustainable strategies for the valorisation of carbohydrate-rich feedstocks.

## Figures and Tables

**Table 1:** Catalyst screening in glucose-to-fructose isomerization reaction in water medium (reaction time = 30 min, VH_2_O = 5 mL, m(glucose) = 225 mg, m(catalyst) = 30 mg).

Catalyst	Temperature	Glucose Conv.	Fructose Yield	Selectivity
AlCl_3_∙6H_2_O	70 °C	-	-	-
PAC	70 °C	-	-	-
Al_13_	70 °C	-	-	-
AlCl_3_∙6H_2_O	90 °C	2.7 ± 0.1	2.5 ± 0.1	94.8 ± 1.9
PAC	90 °C	4.2 ± 0.2	4.1 ± 0.1	99 ± 1.0
Al_13_	90 °C	5.9 ± 0.3	5.8 ± 0.3	98.3 ± 2.0
AlCl_3_∙6H_2_O	120 °C	13.4 ± 0.7	10.2 ± 0.5	76.5± 1.8
PAC	120 °C	14.8 ± 0.7	13.5 ± 0.7	91.3 ± 2.3
Al_13_	120 °C	66.2 ± 3.3	10.4 ± 0.5	15.6 ± 0.4
AlCl_3_∙6H_2_O	130 °C	37.9 ± 1.9	29.9 ± 1.5	78.8 ± 2.0
PAC	130 °C	34.6 ± 1.7	28.9 ± 1.4	83.4 ± 2.0
Al_13_	130 °C	40.9 ± 2.0	30.9 ± 1.5	75.7 ± 1.8

**Table 2:** T1 measurements at different reaction conditions.

	H_2_O:MeOH %	T1_H_2_0 (r.t.)	T1_CH_3_ (r.t.)	T1_glu (r.t.)	T1_glu (90°)	T1_glu (120°)
Without catalyst	100	2.82	-	1.57	1.46	1.52
	50	1.68	2.88	0.43	0.46	0.48
AlCl_3_∙6H_2_O	100	2.52	-	1.53	1.51	0.98
	50	1.35	2.97	0.35	0.4	0.42
PAC	100	2.54	-	1.48	0.9	0.8
	50	1.49	3.26	0.36	0.33	0.2

**Table 3:** Catalyst screening on real matrix (reaction time = 2 h, m(matrix) = 225 mg, m(catalyst in solution) = 30 mg).

Catalyst	Solvent	Conv.	Fructose Yield	Selectivity
AlCl_3_∙6H_2_O	H_2_O	35.6 ± 1.7	25.6 ± 1.2	71.9 ± 2
PAC	H_2_O	23.9 ± 1.2	20.6 ± 1.0	85.8 ± 2.5
AlCl_3_∙6H_2_O	H_2_O: MeOH 1:1	63.8 ± 2.9	53.9 ± 2.5	84.6 ± 2.5
PAC	H_2_O: MeOH 1:1	62.5 ± 3.1	61.1 ± 3.0	97.8 ± 2.9

## Data Availability

The raw data supporting the conclusions of this article will be made available by the authors on request.
